# Geodetector analysis of individual and joint impacts of natural and human factors on maternal and child health at the provincial scale

**DOI:** 10.1038/s41598-024-52282-2

**Published:** 2024-01-18

**Authors:** Jialu Chen, Shuyuan Wang, Ying Han, Yongjin Zhang, Yuansheng Li, Beibei Zhang, Xiang Li, Junhui Zhang

**Affiliations:** Department of Epidemiology and Health Statistics, School of Public Health, Southwest Medical University, No.1, Section 1, Xianglin Road, Longmatan District, Luzhou, 646000 Sichuan People’s Republic of China

**Keywords:** Ecological epidemiology, Health policy, Health services, Public health, Environmental social sciences

## Abstract

This ecological study examined the individual and joint impacts of natural–human factors on the spatial patterns of maternal and child health status in China at the provincial scale in 2020. We considered natural factors (forest coverage, average temperature, and total sulfur dioxide and particulate matter emissions) and human factors (economic development, urbanization, healthcare access, and education level). We combined maternal, infant, and under-five mortality rates into a composite maternal and child health index using the entropy method. The spatial autocorrelation analysis of this index highlighted distinct health patterns across provinces, whereas the geodetector method assessed the effects of natural–human factors on the patterns. A notable east–central–west stepwise decline in health status was observed. Global Moran’s *I* showed positive spatial clustering, with high–high clustering areas in the Yangtze River Delta and low–low clustering areas in western regions. Factor detection identified eight significant natural–human factors impacting maternal and child health, with total sulfur dioxide emission density having the greatest impact. The interaction between average schooling years and total sulfur dioxide emission notably affected maternal and child health patterns. The study concludes that natural–human factors critically affect the spatial distribution of maternal and child health.

## Introduction

The core indicators of maternal and child health (MCH) include maternal mortality rate (MMR), infant mortality rate (IMR), and under-five mortality rate (U5MR) (hereinafter referred to as the “three-rate indicators”). These indicators are important evaluation criteria for measuring the social and economic development levels of a country or region and are also the core evaluation indicators of the United Nations’ sustainable development goals^1^. The global MMR and IMR have declined in the past few decades due to the rapid development of the global economy and improvements in medical and healthcare levels^2^. However, MCH status in different continents varied significantly due to economic, population, and cultural factors. For example, the MCH status in sub-Saharan Africa is the weakest, while North Africa, Oceania, South Asia, and Southeast Asia are at a moderate level. Comparatively, the status of MCH in Europe, Latin America, and North America is higher^3^.

The three-rate indicators were also included in the Outlines of Development Plans for Women and Children and the Healthy China 2030 ^4^. Under the guidance of these plans and with significant improvements in medical and healthcare levels in recent years, China’s commitment to MCH has experienced a substantial leap forward, and MCH status has been significantly improved^5^. According to the “China Statistical Yearbook 2021”^6^, as of 2020, the MMR, IMR, and U5MR had all declined significantly nationwide, decreasing by 15.9%, 33.3%, and 30%, respectively, compared with 2015. Currently, the core indicators of MCH in China are lower than the average level of middle-income countries, and the MMR is lower than the median of all countries globally. However, there were regional imbalances in China’s economic and social development and differences in MCH services, leading to significant variations in the MCH status in different regions. These regional differences were the result of the comprehensive influence of various natural and human factors, the influencing mechanism of which was very complex. Therefore, it is necessary to develop an in-depth understanding of the main factors affecting MCH and their interactions, narrow the gap in MCH between China’s regions, and promote the improvement of MCH status nationwide, which will contribute to the smooth implementation of the “Healthy China 2030” strategy.

Various studies have investigated the factors influencing MCH in China, indicating that both human factors, such as economic development level, urbanization level, accessibility to health services, cultural and educational level^7–9^, and environmental factors, such as temperature^10^ and air pollution^11^, specifically total sulfur dioxide (SO_2_)^12^ and particulate matter (PM)^13^ emissions, play significant roles. However, the interaction between natural and human factors and their impact on MCH remains underexplored. Traditional analysis methods, such as Pearson correlation analysis^14^, multiple linear regression models^15^, multilevel models^16^, and spatial econometric models, such as spatial regression models and geographic weighted regression models^17^, have been employed to study these impacts on MCH. These methods, while useful, often face challenges with multicollinearity among variables, limiting their effectiveness. In response to this limitation, our study introduces the novel application of the geodetector method, which uniquely overcomes the constraints of linear assumptions and multicollinearity. This method^18^ has gained popularity in health research, demonstrating its effectiveness in elucidating complex health issues. For instance, it has been instrumental in identifying key factors in the transmission of dengue fever in Guangzhou, China^19^, and in analysing the spatial epidemiological characteristics of hand, foot and mouth disease (HFMD) in Shantou, China^20^.

In addition, most studies use single MCH outcome indicators, such as one or two of the three-rate indicators^21–24^, and lack comprehensive research on the three-rate indictors, making it difficult to comprehensively and objectively reflect the true situation of MCH status in different regions. By employing the geodetector method and comprehensive three-rate indicators of MCH, our research offers a fresh perspective on the individual and joint effects of natural–human factors. This unique approach allows for a more detailed and accurate exploration of MCH spatial patterns in China. The insights from this research are expected to provide critical guidance for policymakers and healthcare professionals, informing strategies and interventions to enhance MCH across various regions.

## Methods

### Study area and population

This study focused on mainland China, including 31 provinces (municipalities directly under the central government and autonomous regions), excluding Hong Kong, Macao, and Taiwan. Mainland China extends from 73.66°E to 135.05°E in longitude and from 18.15°N to 53.55°N in latitude. According to the 7th National Population Census of China, the total population of mainland China in 2020 was approximately 1,411,778,724, and the area encompasses approximately 9.637 million square kilometers, representing a diverse range of human and environmental conditions pertinent to our study. Moreover, based on the regional classification criteria provided by the Chinese National Bureau of Statistics, all 31 provinces of mainland China were categorized into eastern, central, and western regions to facilitate analysis^25^.

### Geographic mapping

A map of China’s administrative regions at the provincial scale was obtained from the National Basic Geographic Information Center^26^, based on the 1:1,000,000 administrative division map in 2019 (Albers equal-area conic projection).

### Indicator system and data source

#### Dependent variables: three-rate indicators

In this study, we refer to the “three-rate indicators” as the primary outcome measures of MCH. These three indicators play a crucial role in assessing MCH status within a given population. MMR represents the number of maternal deaths per 100,000 births in a specific region or population within a given time frame. It is a critical indicator of the quality of maternal healthcare and childbirth safety. A lower MMR indicates better maternal healthcare and reduced maternal mortality^27^. IMR measures the number of deaths of infants under one year of age per 1000 births. It is a fundamental indicator of overall health and healthcare access for newborns. A lower IMR reflects better infant healthcare and increased chances of survival for newborns^28^. U5MR calculates the number of deaths of children under 5 years per 1000 births. This indicator provides insights into overall child health and well-being within a population. A lower U5MR indicates better child healthcare and improved survival rates for children under 5 years^29^. Due to the “China Statistical Yearbook” providing only national-level data on the three-rate indicators, and not at the provincial level, the provincial-scale three-rate indicators data had to be obtained through the official government websites of each province. For Jilin Province, Heilongjiang Province, and Tibet, the data were acquired by directly contacting the respective provincial government departments. The data for the remaining 28 provinces used in this study were gathered from the “14th Five-Year Plan for the Development of Health and Health Care” published on the government websites of these provinces. The URLs for accessing these data are accessible from Supplementary Table S1.

#### Independent variables: natural and human factors

This study evaluates the influence of natural and human factors on MCH status. In determining the factors to be included, we conducted a comprehensive review of previous research, which indicated that MCH statuses across regions are influenced by both natural factors (the natural environment and environmental quality) and human factors (economic development, urbanization level, accessibility of health services, and the level of cultural education). To ensure a robust and systematic selection of indicators, we followed a two-step process. First, we identified a broad set of potential factors based on their relevance to MCH outcomes, as presented in the existing literature^7,8,10,11^. Second, we applied criteria for scientific rigor and data availability to narrow down the list to the most pertinent and measurable indicators. This led us to select eight natural–human indicators as independent variables (Table 1). These included four natural factors: the natural environment (forest coverage rate and annual average temperature) and environmental quality (total SO_2_ emissions density and total PM emissions density). Additionally, four human factors were selected: the level of economic development (per capita gross regional product [Per. GRP]), urbanization level (urbanization rate), accessibility of health services (per capita number of health care institutions), and the level of cultural education (average years of schooling in the population). Further, to better estimate the impact of environmental factors, we used the annual average temperature of provincial capital cities as a substitute variable for the provincial natural environment. Moreover, each province’s SO_2_ and PM emissions were divided by its area to obtain the environmental pollution density^30^.

**Table 1:** Potential natural–human factors affecting maternal and child health (MCH) in China.

Dimension	Factor/indicator	Units	Variable ID
Natural factors	Forest coverage rate (FCR)	%	*N*_1_
	Annual average temperature (AATem)	°C	*N*_2_
	Total emission density of SO_2_* (TSO_2_)	ton/10,000 km^2^	*N*_3_
	Total emission density of PM* (TPM)	ton/10,000 km^2^	*N*_4_
Human factors	Per capita gross regional product (Per. GRP)	RMB	*H*_1_
	Urbanization rate (UrbR)	%	*H*_2_
	Per capita number of health care institutions (Per.HCI)	no. per 1000 people	*H*_3_
	Average years of schooling in the population (AYS)	years	*H*_4_

*Total emission densities of SO_2_ and PM were calculated by dividing the total SO_2_ and PM emissions of each province by its area.

In our analysis, the independent variable “average years of schooling in the population” was obtained from “The Bulletin of the Seventh National Population Census”^31^. The remaining natural–human factors were derived from the “China Statistical Yearbook 2021.”

### Calculation of the composite MCH index

In our study, based on existing research^32^, we selected the entropy method using Excel 2022 software to combine MMR, IMR, and U5MR as the composite MCH index for each province to reflect MCH status. This composite MCH index was subsequently used to analyse the spatial patterns and driving factors of MCH status in China. The advantage of the entropy method lies in its objective weighting, which minimizes the impact of subjective bias and more comprehensively reflects each mortality rate indicator's contribution to the overall MCH status^33^. This method's objective weighting of indicators makes it a suitable choice for our analysis, providing a more balanced and nuanced understanding of the factors influencing MCH. The calculation steps are as follows^34^:

Data normalization: The three-rate indicators are processed to be dimensionless. Because these indicators are negatively oriented (indicating a lower value is better), the transformed value ${X}_{ij}^{\prime}$ is calculated using the following formula:

1 $${X}_{ij}^{\prime}=\left(max\left\{{X}_{j}\right\}-{X}^{ij})/(max\left\{{X}_{j}\right\}-min\left\{{X}_{j}\right\}\right)$$

Calculation of proportions: The proportion of province *i* under indicator *j*, which helps in understanding the relative importance of each province in each indicator, is calculated as follows:

2 $${Y}_{ij}=\frac{{X}_{ij}^{\prime}}{\sum_{i=1}^{m}{X}_{ij}^{\prime}}$$

Calculation of information entropy: The information entropy for each indicator is calculated as follows:

3 $${e}_{j}=-k\sum_{i=1}^{m}\left({Y}_{ij}\times {{\ln}}{Y}_{ij}\right),\quad k=\frac{1}{{{\ln}}m},0\le {e}_{j}\le 1$$

Calculate the coefficient of variation (CV) for indicator *j*: The CV directly influences the weight size assigned to an indicator. A larger CV value indicates a greater importance in evaluation, resulting in a larger weight.

4 $${d}_{j}=1-{e}_{j}$$

Determination of weights: The weights are calculated based on the information entropy, where a lower entropy indicates a higher weight.

5 $${w}_{i}=\frac{{d}_{j}}{\sum_{j=1}^{n}{d}_{j}}$$

Calculation of the composite index: The composite MCH index for each province is calculated using the weight and proportion matrix:

6 $${S}_{ij}={w}_{i}\times {X}_{ij}^{\prime}$$

where ${X}_{ij}$ represents the value of indicator* j* for province *i*, $min\left\{{X}_{j}\right\}$ and $max\left\{{X}_{j}\right\}$ denote the minimum and maximum values of indicator *j* among all provinces, respectively, and *m* and *n* denote the number of provinces and indicators, respectively.

During the entropy method calculation, a higher information entropy of a mortality rate indicator results in a lower differential coefficient and weight, suggesting a lesser contribution to the composite factor, and vice versa. The final composite MCH index obtained from this study is a positive indicator, where higher values denote better outcomes. The results are presented in Supplementary Tables S2 and S3 and were used for further spatial autocorrelation and geodetector analyses.

### Description of spatial patterns of MCH status

#### Division method

In this study, the natural break (Jenks) method is used to classify data based on inherent natural groupings^35^. This method effectively minimizes variance within each class and maximizes it between classes for all numerical data, particularly suitable for data with non-normal distributions. In classification, the choice of the number of breaks is critical, with 5, 7, and 9 being common choices. Fewer than five classes may lack detail, whereas more than nine could blur key distinctions^36^. This method was used to classify the composite MCH index of provinces into five, which is the default number in ArcGIS 10.2 (ESRI, Redlands, CA, USA). This helped create spatial distribution maps and enhanced comparisons across provinces. Additionally, for the subsequent geodetector analysis requiring discrete independent variables, this method was similarly used to classify the independent variables into five.

#### Spatial autocorrelation

Spatial autocorrelation is a method used for spatial data analysis, and one of its most used statistical measures is Moran’s *I* index^37^. This index determines whether the spatial distribution structure of data exhibits clustering or randomness and includes both global Moran’s *I* spatial autocorrelation and local Moran’s *I* spatial autocorrelation. The global Moran’s* I* was calculated to measure the degree of global clustering of MCH statuses across the study area. The equation used for calculating global Moran’s *I* was:

7 $$I=\frac{\sum_{i=1}^{n}\sum_{j=1}^{n}w_{ij}(x_{i}-\bar{x})(x_{j}-\bar{x})}{S^{2}\sum_{i=1}^{n}\sum_{j=1}^{n}w_{ij}} $$

where *n* represents the sample size, which in this study was the 31 provinces of China. $xi$ represents the value of the MCH index for province *i*, $xj$ represents the value of the MCH index for province *j*, $\overline{x}$ represents the average value of the MCH index for all provinces in China, and* S*^2^ represents the variance of the MCH index for province *i*. $wij$ is the spatial weight between the *i*-th and* j*-th units, and the queen adjacency matrix was used to determine binary adjacency weights. Moran’s, *I* value range from − 1 to 1, where a zero value indicates randomness, positive indicates clustering (stronger with larger values), and negative indicates heterogeneity (stronger with greater absolute values) in MCH.

Global Moran’s *I* hypothesis test used a *Z*-test with* Z* ≥ 1.96, indicating the presence of global spatial autocorrelation or clustering of MCH statuses among provinces (*p* ≤ 0.05), and *Z* < 1.96, indicating no global spatial autocorrelation or random distribution of MCH statuses among provinces (*p* > 0.05).

Local Moran’s *I* index was an indicator that measured the similarity between observation values in a local area and neighboring areas, and effectively identified the local spatial clustering patterns and discrete characteristics of MCH status and classified them into four aggregation types: high–high, low–high, low–low, and high–low. The calculation equation is:

8 $$I_{i}=\frac{(x_{i}-\bar{x})}{S^{2}}\sum_{j}w_{ij}(x_{j}-\bar{x})$$

The hypothesis of local Moran’s* I* used a *Z*-test, with *Z* ≥ 1.96 (*P* ≤ 0.05), indicating positive spatial correlation (high–high or low–low clustering), and* Z* < 1.96 (*P* > 0.05), indicating spatial negative correlation (high–low or low–high outliers) among MCH statuses between provinces.

Geoda 1.12.1 software (Luc Ancelin at UChicago) was used to perform spatial autocorrelation analysis.

#### Geodetector

Geodetector developed by Wang et al.^38^ is a sophisticated method used to detect spatial differentiation and reveal its driving forces. This method operates on the principle that if a variable had a significant impact on MCH, then the spatial pattern of that variable should be similar to that of the MCH. A key advantage of the geodetector method is its non-reliance on linear assumptions and resilience to multicollinearity, making it suitable for socioeconomic and health research. In addition, it is effective even with small sample sizes, bypassing the need for large sample units often required by traditional statistical methods^18^. Distinctively, this method includes four key components: factor detection, interaction detection, risk detection, and ecological detection.

This study used factor detection and interaction detection to assess the individual and joint impacts of natural–human factors on MCH. Factor detection evaluates the individual impact of each factor on MCH status by calculating its *q* value, which ranks the contribution in terms of influence. The equation for calculating the *q* value of factor detection was:

9 $$9 q=1-\frac{\sum_{h=1}^{L}N_{h}\sigma_{h}^{2}}{N\sigma^{2}}=1-\frac{SSW}{SST}$$

10 $$SSW=\sum_{h=1}^{L}N_{h}\sigma_{h}^{2}, \quad SST=N\sigma^{2}$$

where *h* = 1, 2, …, *L* represents the stratification of the composite MCH index or their drivers; *N*_*h*_ represents the number of units in layer *h*, and *N* represents the total number of units in the region. *SSW* is the sum of variances within layers, and *SST* is the total variance of the entire region. In the stratification generated by the independent variables, the range of *q* values is between 0 and 1. A higher *q* value indicates that the driving factor has greater explanatory power of MCH status.

Interaction detection assesses how two or more factors jointly influence the dependent variable, similar to factor detection but including multiplicative interactions. This is particularly significant in our study, as it allows us to understand the complex interactions between socioeconomic and natural factors influencing MCH. This study used two-way interaction detection to analyse the impact of the interaction between natural and human factors on MCH. The resulting *q* value of the interactive factor is used to characterize the relationship between the two factors and their joint impact on MCH. The interaction detection results included five types of interactions: nonlinear weakening, univariate nonlinear weakening, bivariate enhancement, independent, and nonlinear enhancement. The rules for determining the types of interactive effects are shown in Table 2^18^.

**Table 2:** Two-way interactions between potential natural–human factors affecting maternal and child health (MCH).

Interaction	Strength	Type of modeling
*q*(*X*_1_ ∩ *X*_2_) < Min [*q*(*X*_1_), *q*(*X*_2_)]	Weak	Nonlinear
Min [*q*(*X*_1_), *q*(*X*_2_)] < *q*(*X*_1_ ∩ *X*_2_) < Max [*q*(*X*_1_), *q*(*X*_2_)]	Weak	Univariate
*q*(*X*_1_ ∩ *X*_2_) > Max [*q*(*X*_1_), *q*(*X*_2_)]	Strong	Bivariate
*q*(*X*_1_ ∩ *X*_2_) = *q*(*X*_1_) + *q*(*X*_2_)	Independent	
*q*(*X*_1_ ∩ *X*_2_) > *q*(*X*_1_) + *q*(*X*_2_)	Strong	Nonlinear

*X*_1_ and *X*_2_ represent natural and human factors, respectively; *q*(*X*_1_) and *q*(*X*_2_) represent the *q* value of variables *X*_1_ or* X*_2_, respectively; and *q*(*X*_1_ ∩ *X*_2_) represents the *q* value of the interaction between variables *X*_1_ and *X*_2_. Min [*q*(*X*_1_), *q*(*X*_2_)] and Max [*q*(*X*_1_), *q*(*X*_2_)] represent the minimum and maximum values of the q values of *X*_1_ and *X*_2_, respectively.

### Ethics approval

Permission for data access was obtained from the provincial health commissions, the National Bureau of Statistics through an online request to the China Statistical Yearbook 2021 (http://www.stats.gov.cn/sj/ndsj/2021/indexeh.htm) and the Bulletin of the Seventh National Population Census (https://www.gov.cn/guoqing/2021-05/13/content_5606149.htm). The data in this study are publicly available without any personal identifying information.

### Map disclaimer

The depiction of boundaries on this map does not imply the expression of any opinion whatsoever on the part of Scientific Reports (or any member of its group) concerning the legal status of any country, territory, jurisdiction, or area or of its authorities. This map is provided without any warranty of any kind, either express or implied.

## Results

### Spatial distribution of MCH based on the composite index

**Figure 1:**
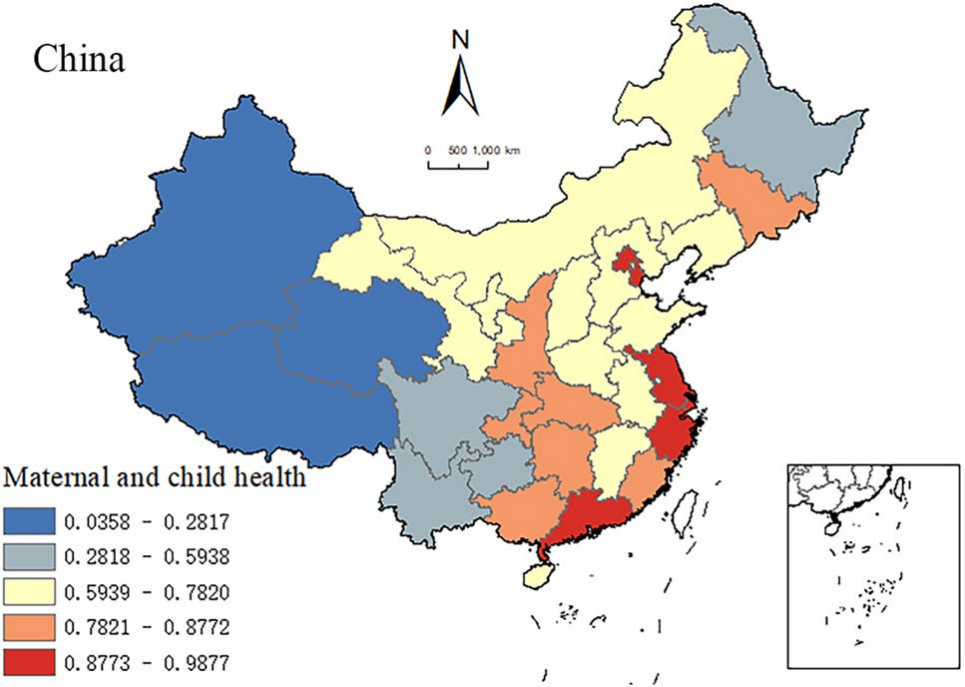
Interprovincial spatial distribution of maternal and child health (MCH) status in China in 2020. Note: This map is based on publicly available data from the National Geographic Information Resources Catalog Service System of China. For more information, visit www.webmap.cn. The inset map offers a detailed representation of the South China Sea Islands.

As depicted in Fig. 1, the spatial distribution of MCH statuses showed an imbalance with a notable stepwise decline in the east–central–west pattern, consistent with the provincial spatial distribution patterns reflected by the three mortality rate indicators (Supplementary Fig. 1). This consistency validates the use of the composite MCH index as a comprehensive indicator for assessing MCH levels in each province. The regions with the highest MCH index values were located in the southeast coastal areas of Shanghai, Jiangsu, Zhejiang, Guangdong, Beijing, and Tianjin, where the values ranged from 0.8773 to 0.9877. Shaanxi, Hubei, Chongqing, Hunan, and Guangxi recorded moderately high MCH index values ranging from 0.7821 to 0.8772. In contrast, the western provinces of Xinjiang, Tibet, Qinghai, Sichuan, Yunnan, and Guizhou had the lowest values of the MCH index, ranging from 0.0358 to 0.5938.

### Spatial autocorrelation analysis of MCH status

In 2020, the provincial-scale analysis of MCH status in China revealed a statistically significant positive global spatial correlation (Moran’s *I* = 0.557, *p* = 0.001), as indicated by the results of the global spatial autocorrelation analysis.

**Figure 2:**
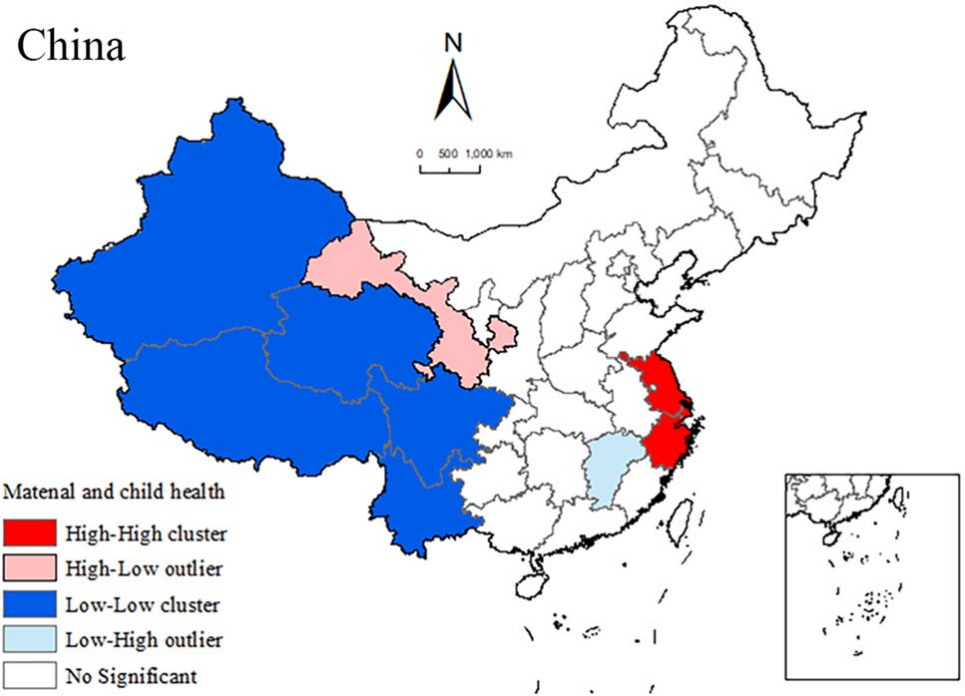
LISA cluster map of maternal and child health (MCH) status at the provincial scale in China in 2020. Note: This map is based on publicly available data from the National Geographic Information Resources Catalog Service System of China. For more information, visit www.webmap.cn. The inset map offers a detailed representation of the South China Sea Islands.

Figure 2 displays the LISA clustering map of MCH statuses in China, highlighting distinctive spatial distribution characteristics of high–high clustering in the east and low–low clustering in the west. Specifically, high–high clustering areas were identified in the Yangtze River Delta regions of Shanghai, Jiangsu, and Zhejiang. In contrast, low–low clustering areas were observed in the western regions of Xinjiang, Tibet, Qinghai, Sichuan, and Yunnan. Additionally, Jiangxi was identified as the low–high outlier area, whereas Gansu was the high–low outlier area.

### Spatial distribution of natural–human factors

**Figure 3:**
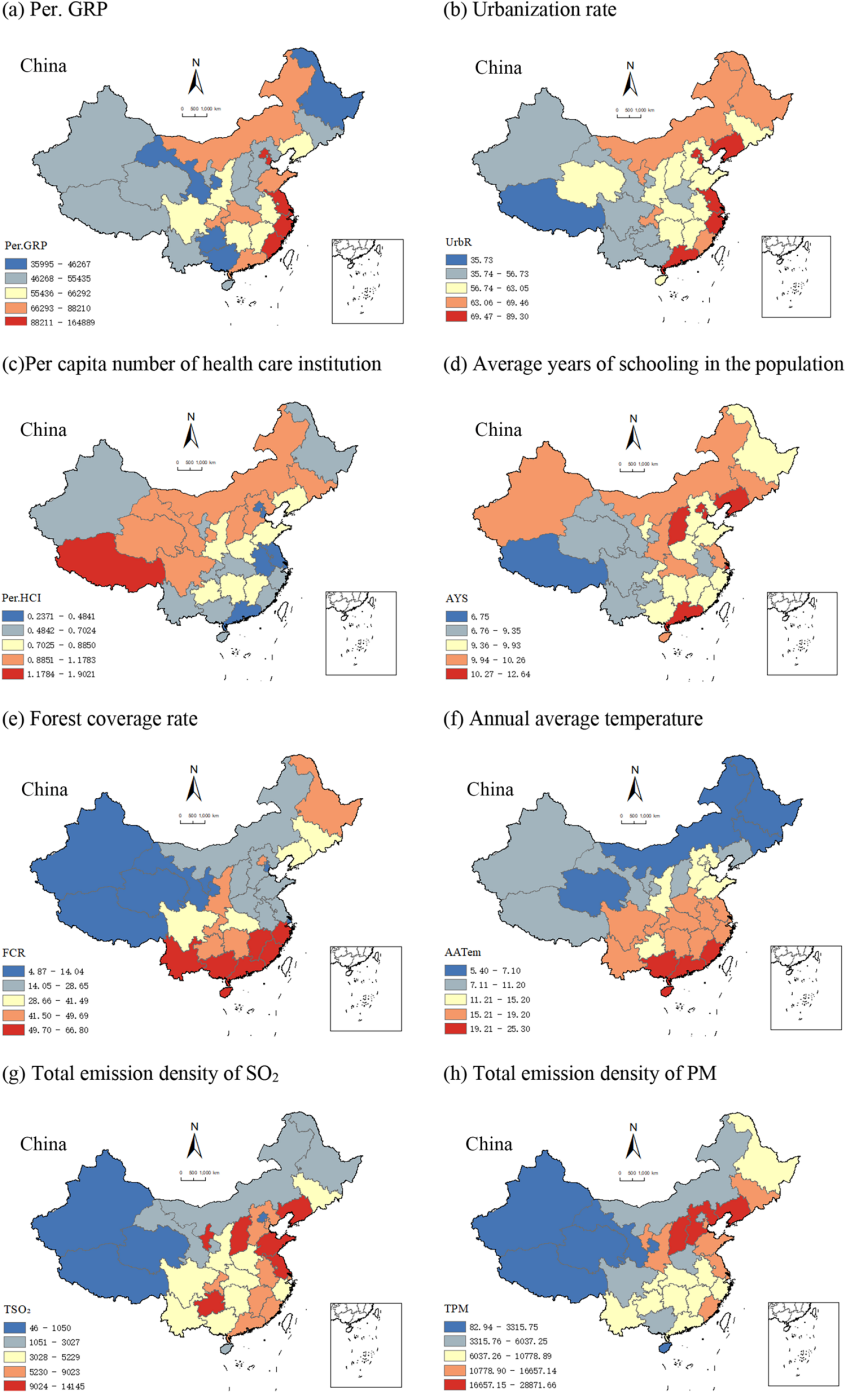
Spatial distribution of natural–human factors in China in 2020. (**a**) Per capita gross regional product (Per. GRP); (**b**) Urbanization rate (UrbR); (**c**) Per capita number of health care institutions (Per. HCI); (**d**) Average years of schooling in the population (AYS); (**e**) Forest coverage rate (FCR); (**f**) Annual average temperature (AATem); (**g**) Total emission density of SO_2_ (TSO_2_); (**h**) Total emission density of PM (TPM). Note: This map is based on publicly available data from the National Geographic Information Resources Catalog Service System of China. For more information, visit www.webmap.cn. The inset map offers a detailed representation of the South China Sea Islands.

Figure 3 depicts the spatial distribution of the eight natural–human factors that may potentially impact MCH status. Per. GRP (Fig. 3a) and the urbanization rate (Fig. 3b) exhibited an overall trend of stepwise decline from southeast to northwest. In contrast, the per capita number of health care institutions (Fig. 3c) did not reveal any discernible relationship with the spatial distribution of MCH status. Average years of schooling in the population (Fig. 3d) were high in the east and low in the west. Forest coverage rate (Fig. 3e) demonstrated a decreasing trend from the southern and northeastern regions to the west, while annual average temperature (Fig. 3f) decreased from south to north. Both the total emission density of SO_2_ (Fig. 3g) and the total emission density of PM (Fig. 3h) showed an overall decreasing trend from southeast to northwest.

### Individual effects on MCH status

The factor detector analysis demonstrates significant impacts of all eight natural–human factors on MCH status (*p* < 0.05), with varying degrees of influence as reflected by their *q* values. As depicted in Table 3, the total emission density of SO_2_, with the highest *q* value (0.8305), emerges as the most influential factor, followed by the total emission density of PM (*q* = 0.6973), highlighting the severe impact of air pollution on MCH. The forest coverage rate (*q* = 0.6704) indicates the positive effects of natural environments on MCH. Healthcare accessibility, represented by the per capita number of healthcare institutions (*q* = 0.6334), and urbanization rate (*q* = 0.5983) emphasize the influence of infrastructure and urban development. Education level (average number of years of schooling in the population, *q* = 0.5727) and economic conditions (Per. GRP, *q* = 0.5585) also significantly affect MCH. The annual average temperature (*q* = 0.4604) suggests environmental conditions’ role.

**Table 3:** The *q* values of factor detection for natural–human factors affecting maternal and child health (MCH).

Driving factors	*q* value	Driving factors	*q* value
Forest coverage rate	0.6704	Per. GRP	0.5585
Annual average temperature	0.4604	Urbanization rate	0.5983
Total emission density of SO_2_	0.8305	Per capita number of health care institution	0.6334
Total emission density of PM	0.6973	Average years of schooling in the population	0.5727

### Joint effects on MCH status

Table 4 illustrates the interaction between two pairs of natural–human factors and their impact on MCH. A total of 28 pairs of factors had statistically significant interactions (*p* < 0.05), resulting in an enhanced two-factor effect as determined by *q*(*X*_1_ ∩ *X*_2_) > MAX(*q*(*X*_1_), *q*(*X*_2_)), indicating that the combined influence of two factors on MCH was more significant than the expected effect of each individual factor alone.

**Table 4:** The* q* values of joint effects of natural–human factors on maternal and child health (MCH).

Factor	*N*_1_	*N*_2_	*N*_3_	*N*_4_	*H*_1_	*H*_2_	*H*_3_	*H*_4_
*N*_1_	0.6704							
*N*_2_	0.7105	0.4604						
*N*_3_	0.8949	0.8790	0.8305					
*N*_4_	0.7491	0.7579	0.8867	0.6973				
*H*_1_	0.9059	0.7640	0.8884	0.9314	0.5585			
*H*_2_	0.8782	0.7852	0.9536	0.8566	0.8048	0.5983		
*H*_3_	0.8469	0.7503	0.9374	0.8230	0.8577	0.7967	0.6334	
*H*_4_	0.8942	0.9660	0.9743	0.8980	0.8638	0.9554	0.8941	0.5727

*N*_1_ represents the forest coverage rate; *N*_2_ represents the annual average temperature; *N*_3_ represents the total emission density of SO_2_; *N*_4_ represents the total emission density of PM; *H*_1_ represents the per capita gross regional product; *H*_2_ represents the urbanization rate; *H*_3_ represents the per capita number of health care institutions; and *H*_4_ represents the average years of schooling in the population.

The results of the two-way interaction detection revealed that 19 of the 36 pairs of interactive factors had *q* values greater than 0.8, accounting for 52.78%, indicating that more than half of the 2-way interactions could well explain the interprovincial spatial distribution differences in MCH. The top three interactive factors ranked by descending order of *q* values were: average years of schooling in the population ∩ total SO_2_ emission density (0.9743), average annual temperature ∩ average years of schooling in the population (0.9660), urbanization rate ∩ average years of schooling in the population (0.9554).

## Discussion

The present study used the entropy method to develop a composite MCH index and spatial analysis methods to investigate the individual and joint effects of natural–human factors on the spatial patterns of MCH status in China. The findings indicate that the spatial distribution of MCH statuses across provinces was imbalanced in China in 2020, with a general pattern of an east–central–west stepwise decline. Moreover, regions characterized by high values of the MCH index were mainly located in southeastern coastal areas and economically developed areas such as Beijing and Tianjin. Conversely, regions with low values of the composite MCH index were primarily distributed in less economically developed regions, particularly the western region. These results suggest that there is still room for improvement in China’s MCH status.

Factor detection found that all eight natural–human factors were statistically significant. Of these, the total emission density of SO_2_ and PM had the greatest explanatory power, with each showing an overall decreasing trend from southeast to northwest consistent with the spatial distribution pattern of MCH status. This indicated that the two factors were positively associated with MCH status, which contradicts the conclusions drawn by other studies. For instance, Lin et al.^39^ discovered that higher concentrations of SO_2_ and PM_10_ were linked to an increased risk of maternal emotional stress during pregnancy. This could be attributed to the fact that both the total emission density of SO_2_ and PM indirectly reflect the economic status of a region. Economic growth might have significant positive impacts on public health and compensate for the negative effects of atmospheric pollution on MCH, for instance, facilitating better quality healthcare services and providing more healthcare resources to the public^40^.

The explanatory power of the forest coverage rate ranked third, showing a positive association with MCH status. This was supported by the geographical pattern observed, with the third-ranking explanatory factor exhibiting a decline from the southern and northeastern regions toward the west, corresponding to the spatial distribution of MCH status. High levels of forest coverage typically indicate a healthier and more visually appealing natural environment, with exposure to nature having the potential to benefit the physical and mental well-being of mothers and children^41,42^.

The per capita number of healthcare institutions did not exhibit a spatial pattern related to MCH status. Previous studies have suggested a positive association between the per capita number of healthcare institutions and the level of health^43,44^. However, our study found that the Xinjiang Uygur Autonomous Region had the highest per capita number of healthcare institutions but the lowest MCH index value. This could be because most primary healthcare facilities in the Xinjiang Uygur Autonomous Region were village health offices, as reported in the China Statistical Yearbook 2021 (National Bureau of Statistics of China, 2021). Consequently, MCH resources were insufficiently allocated. Moreover, another study revealed the problem of the inequitable distribution of medical and healthcare institutions across different areas in the Xinjiang Uygur Autonomous Region^45,46^. Therefore, optimizing the allocation of primary healthcare institutions, particularly those related to MCH, is essential in this province.

The urbanization rate exhibited a decreasing trend in an east–central–west pattern like the spatial distribution of MCH status in China, suggesting a potential positive association between Per. GRP and MCH status. This indicator reflects the level of urbanization, which results in fundamental changes in social structure, family relations, and lifestyles^47,48^ and holds the potential to improve some MCH problems in developing countries^49^. Additionally, urbanization is typically accompanied by improved health services and health-related knowledge, resulting in better access to healthcare and higher levels of MCH status for urban populations compared with rural populations^50^. It is worth noting that although Liaoning province had a high rate of urbanization, its MCH status was at only a moderate level. This could be linked to a high total emission density of SO_2_ and PM, which led to more severe environmental pollution in Liaoning. Hence, it was crucial to pay attention to the quality of urbanization and take measures to mitigate the negative health effects of urbanization even while accelerating urban development. Moreover, environmental regulations must be strengthened to diminish environmental pollution and enhance living standards in both urban and rural areas.

Per. GRP showed an overall spatial distribution pattern of a stepwise decline from the southeast to the northwest, similar to MCH status, indicating a positive association between Per. GRP and MCH status. The positive association could be a result of regions with higher levels of economic development having better healthcare facilities, more abundant healthcare resources, and higher quality healthcare services^51^. Furthermore, greater economic development was usually accompanied by higher levels of health literacy and education, fostering disease prevention and risk reduction awareness. Overall, economic development was crucial in promoting MCH status and reducing maternal and neonatal mortality^52^.

The average years of schooling in the population showed a stepwise decline in the spatial distribution from east to west, which was similar to the spatial distribution pattern of MCH status, implying a positive association between the variables. This was similar to the results of other studies^53^. Parents with more education were more likely to possess better knowledge about health care and maternal and child nutrition, engage in healthy behavior and provide a hygienic and safe environment for their children, all improving MCH status. However, it is crucial to note that despite higher average years of schooling in the population, some regions, such as Xinjiang, Inner Mongolia, and Heilongjiang, had a lower MCH status. This suggested the need to improve the health literacy levels and health-related knowledge development among residents in these areas.

The results of the two-way interaction detection analysis showed that the influence of the interaction between each pair of driving factors was significantly stronger than that of individual factors, indicating that the spatial heterogeneity of MCH status in China was caused by the nonlinear coupling of multiple factors. The most influential interactive factor on the spatial heterogeneity of MCH status was the combination of average years of schooling in the population and total emission density of SO_2_, highlighting the dominant role of cultural education and environmental pollution in shaping the pattern of spatial heterogeneity of MCH statuses across 31 provinces in China after considering spatial overlapping. The level of environmental pollution not only reflected the degree of air pollution in a region but also indirectly reflected the region’s economic development level. A high economic level could positively influence both the MCH and the educational levels of residents, which, in turn, may positively impact MCH status. Therefore, when average years of schooling in the population and total emission density of SO_2_ worked together, their joint effect on MCH status was enhanced, and the impact of the total emission density of SO_2_ on MCH status was indirect.

Both the annual average temperature and urbanization rate had a strong interaction with average years of schooling in the population on MCH. The annual average temperature had the lowest individual impact on MCH status, with an overall decreasing trend from south to north and little association with the spatial distribution of MCH status. In contrast, urban residents living in cities with high urbanization rates had easier access to health services and improved health knowledge and benefited from a higher average year of schooling in the population due to the overall availability of better educational resources and high-quality talent. Thus, they worked together to promote the improvement of MCH. Generally, the interactive factors with a higher interactive impact were mainly average years of schooling in the population and total SO_2_ emission density. This indicated that the key interactive factors containing either of these two factors had a more significant superposition effect on the spatial differentiation pattern of MCH status in China.

In alignment with China’s significant advancements in MCH under the Healthy China 2030 initiative and the Outlines of Development Plans for Women and Children, the following recommendations are proposed: strengthening environmental protection and pollution control, especially reducing SO_2_ and PM emissions, to improve MCH status. Concurrently, there should be a focus on integrating economic development with enhancements in healthcare quality, particularly in economically underdeveloped areas. In addition, increasing educational investments to elevate health literacy, especially in urbanization processes, is vital along with maintaining environmental sustainability and ecological balance. These actions are designed to further progress in reducing maternal, infant, and under-five mortality rates, thereby supporting the ongoing commitment to MCH and advancing the “Healthy China 2030” strategy. By doing so, China’s efforts can significantly contribute to the global pursuit of the United Nations’ sustainable development goals, showing the close connection between regional health plans and global health outcomes.

To the best of our knowledge, this study is the first to use the geodetector method for a nuanced analysis of individual and joint effects of natural–human factors on MCH status in China. This method uniquely enables us to understand the complex interplay of these factors, a task unachievable with traditional analytical tools. By incorporating a set of three comprehensive indicators (the composite MCH index), we offer a more comprehensive and accurate depiction of MCH across regions, thereby addressing a critical gap in existing research and establishing a new foundation for future studies in this field. However, our focus was limited to provincial-level data in 2020. Future studies could expand this to city or county levels and incorporate spatiotemporal analysis to explore dynamic changes. As an ecological study, our findings may have limited applicability to smaller spatial units and individual-level variations. Thus, we recommend using more diverse data, such as remote sensing data, for broader analysis. Despite these limitations, this study still has important scientific and policy implications.

## Conclusions

This study assessed the conditions of MCH across 31 provinces in China in 2020 using three-rate indicators and analysed the individual and joint impacts of natural–human factors on MCH spatial patterns using the geodetector method. The findings indicated significant regional disparities in MCH, with a notable east–central–west stepwise decline. High–high MCH clustering in the Yangtze Delta and low–low clustering in the western region were identified. Among various factors, the total emission density of SO_2_ stood out as a key influencer of MCH, followed by total emission density of PM, forest coverage rate, per capita number of health care institutions, urbanization rate, Per. GRP, average number of years of schooling in the population and annual average temperature. Further, interactive effects among different factors were observed, with the interplay between average years of schooling and SO_2_ emission density showing a substantial impact on MCH patterns. These insights suggest that policymakers should develop region-specific strategies that consider these natural–human factors. Further research at finer spatial scales and enhanced spatiotemporal data collection is suggested for a deeper understanding of the impact of these factors on MCH.

### Supplementary Information

Supplementary Information 1.

Supplementary Information 2.

## Data Availability

Data are available in a public, open access repository and can be freely downloaded from the official websites of the health care commissions of each province in China, the China Health Statistics Yearbook 2021 (http://www.stats.gov.cn/sj/ndsj/2021/indexeh.htm), and the Bulletin of the Seventh National Population Census (https://www.gov.cn/guoqing/2021-05/13/content_5606149.htm). The datasets generated during and/or analysed during the current study are available from the corresponding author upon reasonable request.
