# Supplementary material for: Geodetector analysis of individual and joint impacts of natural and human factors on maternal and child health at the provincial scale
Source: Sci Rep. 2024 Jan 18;14:1643. doi: 10.1038/s41598-024-52282-2 (PMC10796915; doi:10.1038/s41598-024-52282-2)

**Supplementary materials (Supplementary Table S1, Supplementary Table S2, Supplementary Table S3, Supplementary Figure 1)**

***Journal:*** *Scientific reports*

**Title:** Geodetector Analysis of Individual and Joint Impacts of Natural and Human Factors on Maternal and Child Health at the Provincial Scale

**Authors:** Jialu Chen†, Shuyuan Wang†, Ying Han, Yongjin Zhang, Yuansheng Li, Beibei Zhang, Xiang Li, and Junhui Zhang*

**Supplementary Table S1**. URLs for data access from the 28 provinces in the study

| Province | website |
| --- | --- |
| Beijing | http://www.beijing.gov.cn/zhengce/zhengcefagui/202112/t20211229_2575955.html |
| Tianjin | http://wsjk.tj.gov.cn/ZWGK3158/ZCFG6243_1/GZWJ625/202104/t20210401_5415205.html |
| Hebei | http://info.hebei.gov.cn/eportal/ui?pageId=6806152&articleKey=7007707&columnId=6806589 |
| Shanxi | http://www.shanxi.gov.cn/sxszfxxgk/sxsrmzfzcbm/sxszfbgt/flfg_7203/szfgfxwj_7205/202112/t20211231_949961.shtml |
| Inner Mongolia | https://www.nmg.gov.cn/zwgk/zfxxgk/zfxxgkml/202110/t20211026_1915925.html |
| Liaoning | http://wsjk.ln.gov.cn/zfxxgk_146003/fdzdgknr/ghxx/xggh/ |
| Shanghai | https://wsjkw.sh.gov.cn/szfgfxwj1/20210716/de585b8418be48c291d7c51ab5a7585f.html |
| Jiangsu | http://www.jiangsu.gov.cn/art/2021/10/15/art_46144_10075225.html |
| Zhejiang | http://fzggw.zj.gov.cn/art/2021/5/11/art_1229123366_2284161.html |
| Anhui | https://www.ah.gov.cn/public/1681/554109111.html |
| Fujian | http://wjw.fujian.gov.cn/jggk/csxx/ghyxxc/gzdt/202110/t20211020_5746402.htm |
| Jiangxi | http://www.jiangxi.gov.cn/art/2021/10/28/art_393_3700853.html |
| Shandong | http://wsjkw.shandong.gov.cn/zwgk/ghjh/gh/202107/t20210723_3679215.html |
| Henan | http://www.henan.gov.cn/2022/01-21/2386396.html |
| Hubei | http://wjw.hubei.gov.cn/zfxxgk/fdzdgknr/ghxx/202111/t20211123_3878474.shtml |
| Hunan | http://www.hunan.gov.cn/hnszf/xxgk/wjk/szfbgt/202108/t20210830_20411745.html |
| Guangdong | http://www.gd.gov.cn/gdywdt/zwzt/kjssw/zxgh/content/post_3757902.html |
| Guangxi Zhuang | http://www.gxzf.gov.cn/zfwj/zzqrmzfbgtwj_34828/2022ngzbwj/t11211201.shtml |
| Hainan | https://www.hainan.gov.cn/hainan/szfbgtwj/202106/75e46854f4214209b83533146fdb819d.shtml |
| Chongqing | http://www.cq.gov.cn/zwgk/zfxxgkml/szfwj/xzgfxwj/szf/202201/t20220124_10334603.html |
| Sichuan | http://wsjkw.sc.gov.cn/scwsjkw/ghjh/2021/11/19/b553440a25004fec96cf165e52dbbeb0.shtml |
| Guizhou | http://wjw.guizhou.gov.cn/trsigi/collect/view.html?siteId=500663&id=10158 |
| Yunnan | http://ynswsjkw.yn.gov.cn/web/doc/UU163997292039206457 |
| Shaanxi | http://sxwjw.shaanxi.gov.cn/zfxxgk/fdzdgknr/tjxx/202108/t20210825_2188283.html |
| Gansu | http://wsjk.gansu.gov.cn/wsjk/c113482/202111/1893654.shtml |
| Qinghai | http://fgw.qinghai.gov.cn/ztzl/zt2022/sswgh/zxgh/202202/t20220225_80419.html |
| Ningxia | http://wsjkw.nx.gov.cn/zfxxgk_279/fdzdgknr/wstjbg/ |
| Xinjiang Uyghur | http://www.xinjiang.gov.cn/xinjiang/gfxwj/202201/8beeb83976cc4c939fd49e4a35745348.shtml |

**Supplementary Table S2**. Entropy value and weight results of the three-rate indicators. Note: IMR indicate infant mortality rate; U5MR indicates under-five mortality rate; MMR indicates maternal mortality rate.

| **Indicators** | **IMR** | **U5MR** | **MMR** |
| --- | --- | --- | --- |
| Information entropy | 0.01065304 | 0.01059748 | 0.00985374 |
| Coefficient of variation | 0.98934696 | 0.98940252 | 0.99014626 |
| Weights | 0.33323735 | 0.33325607 | 0.33350658 |

**Supplementary Table S3**. Composite maternal and child health (MCH) index calculated based on the entropy method for 31 provinces in China in 2020.

| Province | Composite MCH index | Province | Composite MCH index |
| --- | --- | --- | --- |
| Beijing | 0.987634 | Hubei | 0.685004 |
| Tianjin | 0.912208 | Hunan | 0.773450 |
| Hebei | 0.705912 | Guangdong | 0.765227 |
| Shanxi | 0.706696 | Guangxi Zhuang | 0.837959 |
| Inner Mongolia | 0.745992 | Hainan | 0.837797 |
| Liaoning | 0.781993 | Chongqing | 0.919725 |
| Jilin | 0.852536 | Sichuan | 0.877196 |
| Heilongjiang | 0.582812 | Guizhou | 0.662045 |
| Shanghai | 0.955807 | Yunnan | 0.808301 |
| Jiangsu | 0.909098 | Tibet | 0.515774 |
| Zhejiang | 0.975380 | Shaanxi | 0.528440 |
| Anhui | 0.781211 | Gansu | 0.593840 |
| Fujian | 0.870013 | Qinghai | 0.035847 |
| Jiangxi | 0.987634 | Ningxia | 0.837228 |
| Shandong | 0.912208 | Xinjiang Uyghur | 0.745548 |
| Henan | 0.705912 | – | – |

**Supplementary Figure 1.** Interprovincial spatial distribution of “three-rate indicators” in China in 2020. (a) Maternal mortality rate (MMR); (b) Infant mortality rate (IMR); (c) under-five mortality rate (U5MR). Note: This map is based on publicly available data from the National Geographic Information Resources Catalog Service System of China. For more information, visit www.webmap.cn.


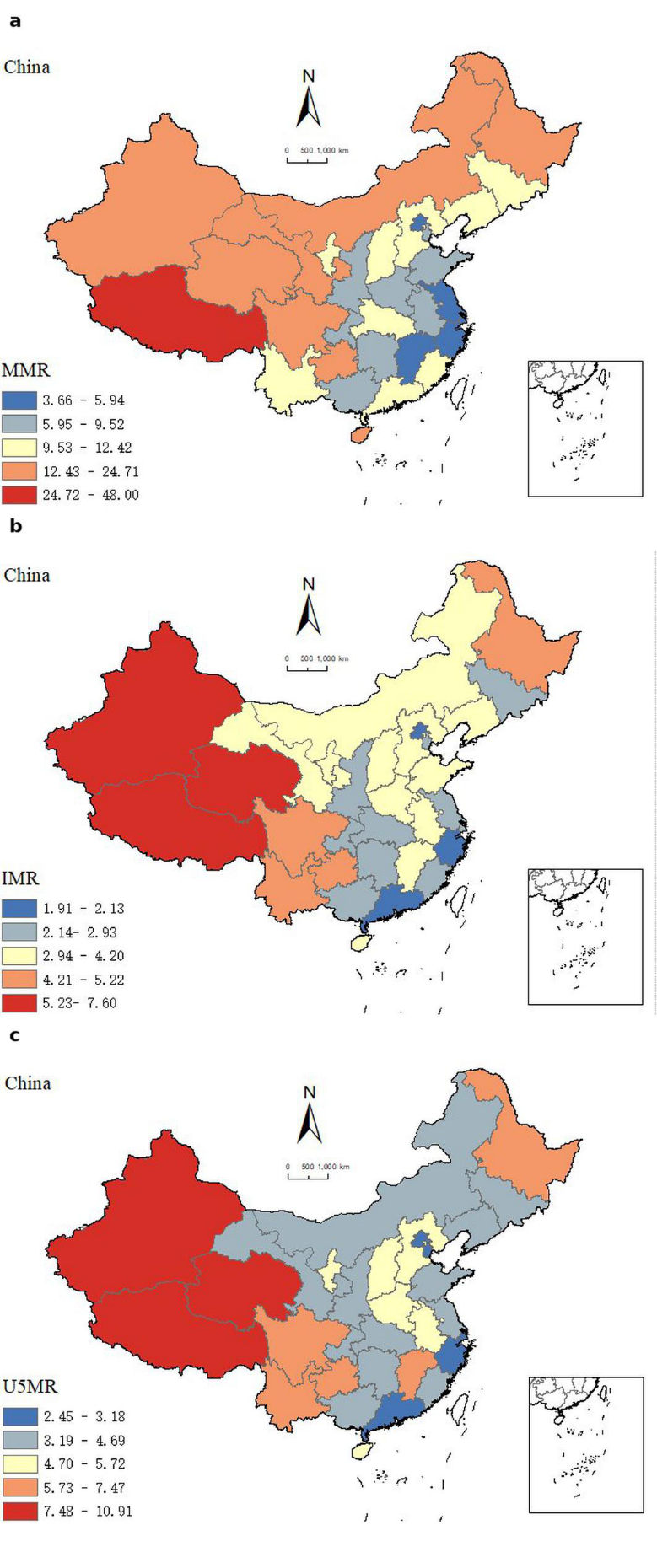

Supplement: Supplementary file 1 — Supplementary Information 1. [file 41598_2024_52282_MOESM1_ESM.docx]
