# Supplementary figures and images for: Geodetector analysis of individual and joint impacts of natural and human factors on maternal and child health at the provincial scale
Source: Sci Rep. 2024 Jan 18;14:1643. doi: 10.1038/s41598-024-52282-2 (PMC10796915; doi:10.1038/s41598-024-52282-2)

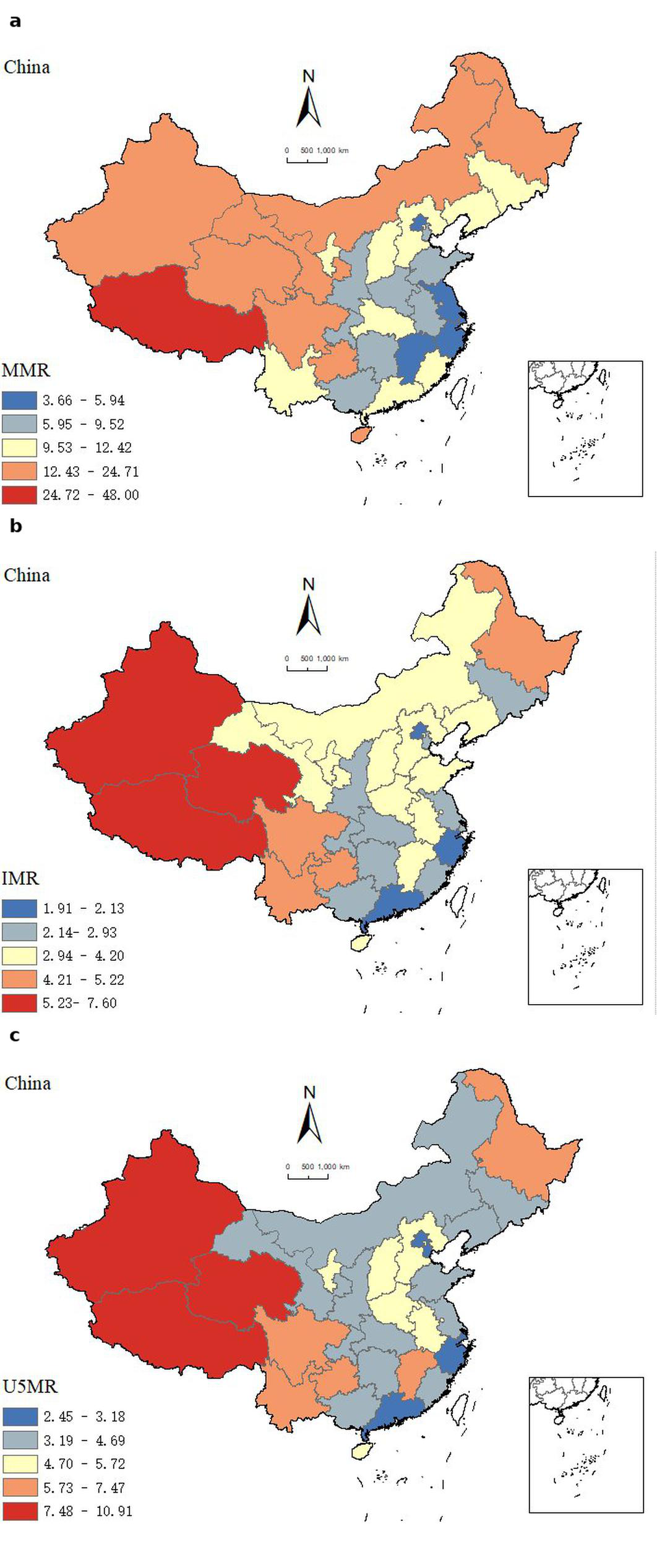

Supplement: Supplementary file 2 — Supplementary Information 2. [file 41598_2024_52282_MOESM2_ESM.tif]
